# Carbon Footprint of Pars Plana Vitrectomy, Pneumatic Retinopexy, and Scleral Buckle Procedures for Rhegmatogenous Retinal Detachment Repair

**DOI:** 10.1177/24741264251367120

**Published:** 2025-09-03

**Authors:** Rahul Moorjani, Graeme Kenneth Loh, Matthew T.S. Tennant, Peter J. Kertes, Parampal S. Grewal

**Affiliations:** 1 Department of Ophthalmology and Visual Sciences, University of Alberta, Edmonton, Alberta, Canada; 2 Department of Ophthalmology and Vision Sciences, University of Toronto, Toronto, Ontario, Canada

**Keywords:** retinal detachment repair, carbon footprint, pars plana vitrectomy, scleral buckle, pneumatic retinopexy

## Abstract

**Purpose:** To quantify the environmental impact of pars plana vitrectomy (PPV), pneumatic retinopexy, and scleral buckle procedures used in rhegmatogenous retinal detachment (RRD) repair. **Methods:** We conducted a life cycle assessment of PPV, pneumatic retinopexy, and scleral buckle procedures. The primary outcome measure was the greenhouse gas emissions associated with each procedure measured in carbon dioxide equivalents. **Results:** The total greenhouse gas emissions produced were 51.10 kg carbon dioxide equivalents for PPV, 2.09 kg carbon dioxide equivalents for pneumatic retinopexy, and 12.57 kg carbon dioxide equivalents for scleral buckle. Emissions related to equipment use (30.07 kg carbon dioxide equivalents) followed by equipment manufacturing (21.00 kg carbon dioxide equivalents) were the main contributors of greenhouse gases in PPV. Emissions related to equipment manufacturing (1.60 kg and 8.51 kg of carbon dioxide equivalents for pneumatic retinopexy and scleral buckle, respectively), followed by equipment use (0.49 kg and 4.05 kg of carbon dioxide equivalents for pneumatic retinopexy and scleral buckle, respectively), were the greatest contributors of greenhouse gases in pneumatic retinopexy and scleral buckle. **Conclusions:** There is a substantial difference in greenhouse gas emissions among PPV, pneumatic retinopexy, and scleral buckle. Quantifying and understanding these differences can inform surgical decision-making.

## Introduction

In 2018, the United Nations Intergovernmental Panel on Climate Change released a report highlighting the urgent need to curb carbon dioxide emissions from human activity to reduce the impact of global warming.^1^ The healthcare sector is responsible for approximately 5% of greenhouse gas emissions worldwide.^2^ Ophthalmology is a high-volume surgical specialty, with retinal procedures comprising a substantial portion. A common pathology requiring prompt surgical intervention is a rhegmatogenous retinal detachment (RRD), with an incidence of 6 to 18 cases per 100 000 population.^3^ Treatment options include pars plana vitrectomy (PPV), pneumatic retinopexy, and scleral buckle. Each procedure has its advantages and disadvantages, but no treatment choice has demonstrated unequivocal superiority.^4–6^ Recent technological advances have led to a major shift toward RRD repair with PPV, especially in North America.^7^ However, the Pneumatic Retinopexy versus Vitrectomy for the Management of Primary Rhegmatogenous Retinal Detachment Outcomes Randomized Trial (PIVOT) demonstrated high success with pneumatic retinopexy in patients meeting specific criteria and showed superior visual outcomes compared to PPV.^6^ Furthermore, scleral buckle demonstrates high primary success rates and continues to play a role in RRD repair.^8^ Given the considerable differences in resource use among PPV, pneumatic retinopexy, and scleral buckle, we aimed to investigate and quantify the carbon footprint associated with each procedure.

## Methods

We collected data from the Royal Alexandra Hospital and the Alberta Retina Consultants outpatient clinic in Edmonton, Alberta, Canada. No individual patient data was collected. All data were collected from PPV, pneumatic retinopexy, and scleral buckle surgeries performed by two staff surgeons (MT and PG). Only uncomplicated PPV cases not involving cataract extraction or general anesthesia were included. PPV and scleral buckle were performed in a hospital-based operating room, while pneumatic retinopexy was performed in an outpatient clinic. Sulfur hexafluoride (SF_6_) was used for gas tamponade in both PPV and pneumatic retinopexy cases. In pneumatic retinopexy, laser retinopexy was applied to retinal breaks within 48 hours of gas injection. Although practice patterns vary, the majority of scleral buckle procedures are performed under general anesthesia, and this was considered in our analysis.^9^ A list of disposable materials and their quantities for each surgery was obtained from the surgeons’ case cards. The raw materials for each instrument were identified through packaging labels or confirmed through discussions with industry representatives. The weight of equipment and total landfill-bound waste was measured directly. No waste was recycled at either facility. Electricity-related emissions were estimated based on the average duration of each procedure, using energy consumption data obtained from the literature and national surveys.^10,11^

A life cycle assessment was conducted to quantify the environmental impact of each procedure, expressed in carbon dioxide equivalents. A life cycle assessment is a tool used to quantify greenhouse gas emissions associated with a process, which in this study was defined as an RRD repair through PPV, pneumatic retinopexy, or scleral buckle. Emissions related to raw material extraction, manufacturing, production, usage, and disposal of instruments and equipment necessary for PPV, pneumatic retinopexy, or scleral buckle were analyzed.^12^ In addition to disposable materials, equipment use accounted for electricity consumption (in both the operating room and office), SF_6_ gas for PPV and pneumatic retinopexy cases, and sevoflurane gas for maintenance of general anesthesia in a portion of scleral buckle cases. The life cycle assessments of other medications were found to be inconsequential and were therefore not included. The scope of our analysis was limited to emissions resulting from perioperative patient care. Pre- and postoperative assessments, as well as travel, were not included in the analysis. Furthermore, the production and disposal of multiuse equipment (eg, vitrectomy machine, operating microscope, etc) were not included in our analysis because of the negligible impact on 1 surgery. A life cycle assessment calculator software (LCA Calculator Ltd) was used to analyze the data. This software uses the internationally recognized emissions database called ecoinvent. To assess the impact of SF_6_, weight was calculated using the ideal gas equation and converted to carbon dioxide equivalents based on the 100-year global warming potential, as reported in the fifth assessment report by the Intergovernmental Panel on Climate Change (Table 1).^13^ Similarly, the amount of sevoflurane used was estimated based on the delivery rate and the duration of the maintenance phase of general anesthesia, then converted to carbon dioxide equivalents based on the 100-year global warming potential.^14^

**Table 1.** Summary of Sulfur Hexafluoride Usage in PPV and Pneumatic Retinopexy Procedures Based on 100-Year Global Warming Potential.

Procedure	Total Volume of SF_6_ (mL)	Volume of Discarded SF_6_ (mL)	Total Emissions (kg Carbon Dioxide Equivalents)
PPV	150	135	22.98
Pneumatic retinopexy	3	2.4	0.46

Abbreviations: PPV, pars plana vitrectomy; SF₆, sulfur hexafluoride.

## Results

**Figure 1.**
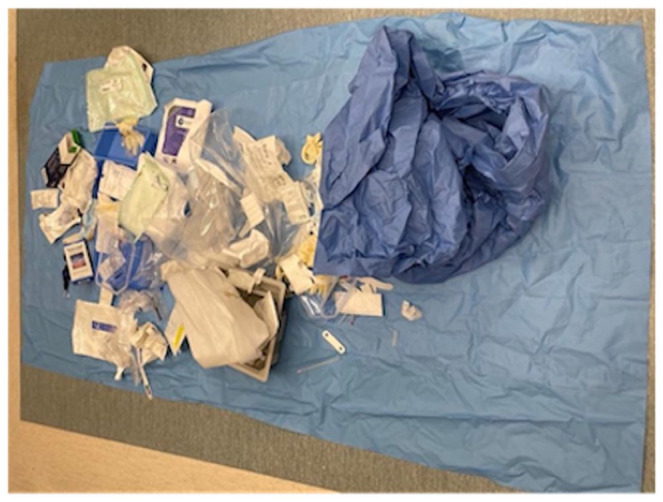
Image of disposable equipment and instruments after rhegmatogenous retinal detachment repair by pars plana vitrectomy.

**Figure 2.**
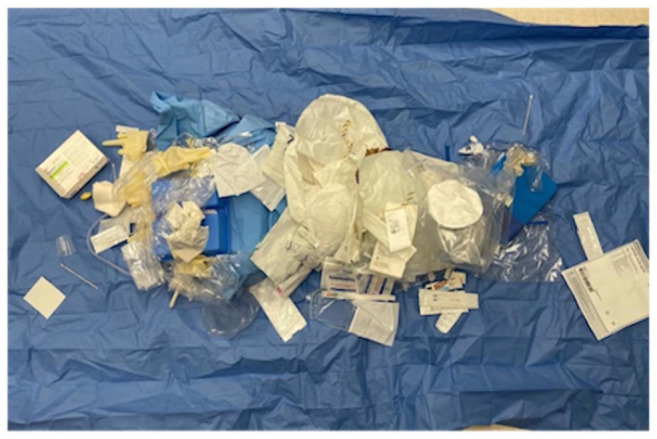
Image of disposable equipment and instruments after rhegmatogenous retinal detachment repair by scleral buckle.

**Figure 3.**
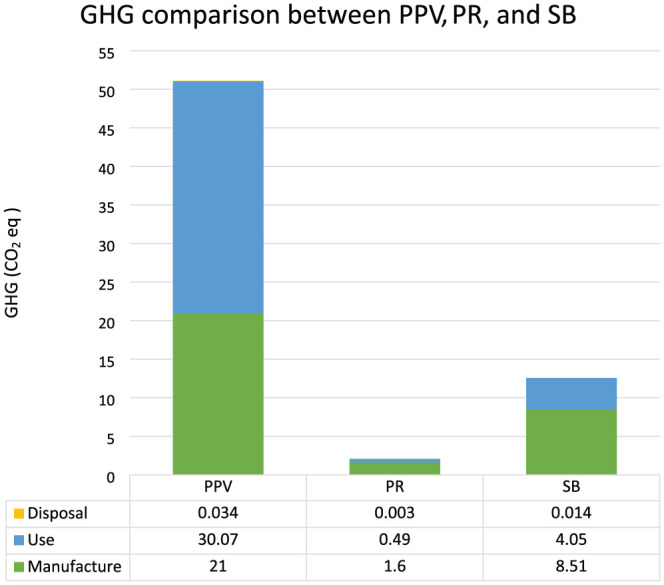
Comparison of greenhouse gas emissions in kg carbon dioxide equivalents produced from pars plana vitrectomy, pneumatic retinopexy, and scleral buckle.

**Figure 4.**
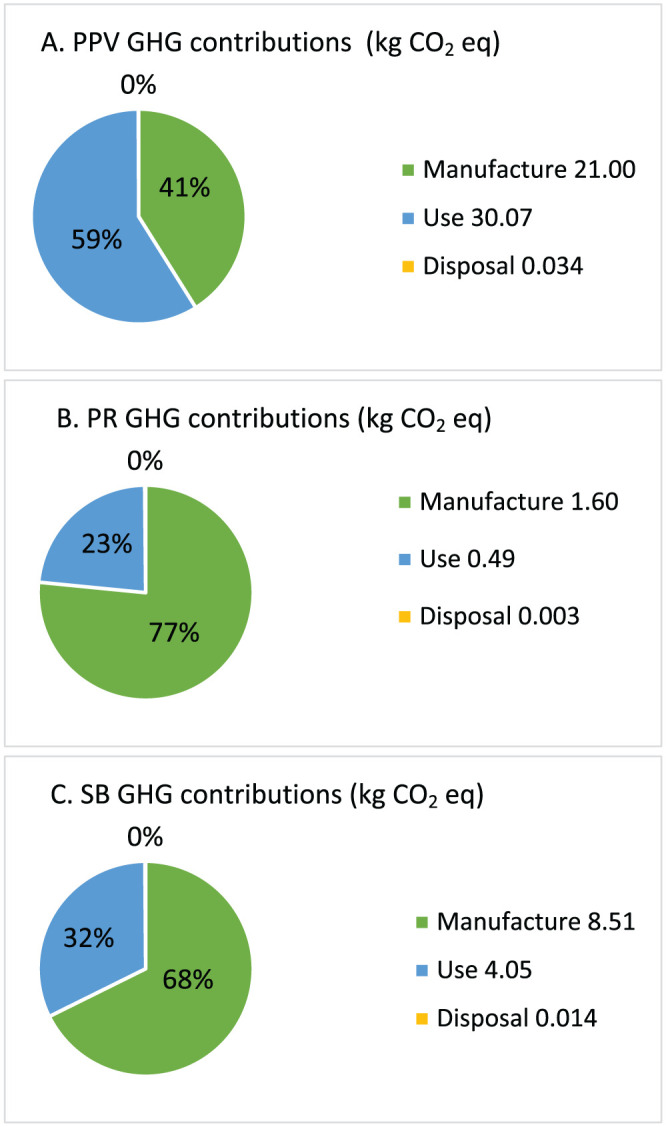
Greenhouse gas emissions in carbon dioxide equivalents from instrument manufacturing, equipment use, and waste disposal for (A) pars plana vitrectomy, (B) pneumatic retinopexy, and (C) scleral buckle. “Manufacturing” includes greenhouse gas emissions from producing disposable items through raw material transformation processes. “Use” covers emissions from equipment operation, including electricity consumption (operating room and office settings), sulfur hexafluoride gas for pars plana vitrectomy and pneumatic retinopexy cases, and sevoflurane gas used in general anesthesia for some scleral buckle cases. “Disposal” refers to emissions from waste sent to a landfill.

The average disposable waste generated per procedure was 2.81 kg for PPV (Figure 1) compared to 0.24 kg for pneumatic retinopexy and 1.19 kg for scleral buckle (Figure 2). The average procedure duration was 71 minutes for PPV, 17 minutes for pneumatic retinopexy, and 46 minutes for scleral buckle. The total greenhouse gas emissions produced by PPV, pneumatic retinopexy, and scleral buckle were 51.10 kg, 2.09 kg, and 12.57 kg of carbon dioxide equivalents, respectively (Figure 3). In PPV cases, the primary contributors to greenhouse gas were equipment use (30.07 kg carbon dioxide equivalents, 59%) and equipment manufacturing (21.00 kg carbon dioxide equivalents, 41%) (Figure 4). For pneumatic retinopexy and scleral buckle, the largest contributors of greenhouse gas emissions were equipment manufacturing, then equipment use (Figure 4). For pneumatic retinopexy, equipment manufacturing emitted 1.60 kg carbon dioxide equivalents (76%), while equipment use contributed 0.49 kg carbon dioxide equivalents (23%). For scleral buckle, equipment manufacturing emitted 8.51 kg carbon dioxide equivalents (68%), while equipment use contributed to 4.05 kg carbon dioxide equivalents (32%) (Figure 4). Among emissions related to equipment use, SF_6_ was a major contributor in both PPV and pneumatic retinopexy, accounting for 22.98 kg (45%) and 0.46 kg (22%) of carbon dioxide equivalents, respectively. The adjusted contribution of sevoflurane gas for scleral buckle cases involving general anesthesia was 0.99 kg carbon dioxide equivalents, representing 8% of total emissions per case. Disposal-related emissions were the smallest contributors across all procedures, accounting for 0.034 kg, 0.003 kg, and 0.014 kg of carbon dioxide equivalents in PPV, pneumatic retinopexy, and scleral buckle, respectively (Figures 3 and 4).

## Conclusions

Our analysis demonstrated that pneumatic retinopexy had the smallest carbon footprint, with emissions from scleral buckle and PPV being approximately 500% and 2300% greater, respectively (Figure 4). Approximately 50 000 RRDs are repaired annually in the United States, and more than 50% of cases are reported to meet PIVOT criteria.^15,16^ If 25 000 RRDs were repaired using pneumatic retinopexy instead of PPV, the resulting difference in greenhouse gas emissions would be 1226 metric tons of carbon dioxide equivalents. To contextualize this difference, it is comparable to the emissions produced by driving 3 000 000 miles in a typical gasoline-powered vehicle, approximately the annual driving distance of 220 average North Americans. A similar comparison between pneumatic retinopexy and scleral buckle would result in a greenhouse gas emission difference of 262 metric tons carbon dioxide equivalents, equivalent to the emissions generated by driving 670 000 miles in a gasoline-powered vehicle or the yearly driving emissions of 50 average individuals.^17^

Emissions related to the manufacturing process of disposable equipment were major contributors of greenhouse gases across all procedures, with a proportionally greater impact observed in pneumatic retinopexy and scleral buckle (Figure 3). From an industry perspective, sustainable product development could be an area of focus for further environmental optimization. Additionally, each disposable instrument is individually packaged in plastic, which contributes to the ecologic impact of disposal in all procedures (Figures 1 and 2). Although disposal-related emissions were relatively low, reconsidering individual packaging while maintaining instrument sterility may help reduce the total volume of waste produced.

The use of SF_6_ for retinal tamponade in PPV and pneumatic retinopexy was a significant contributor to greenhouse gas emissions, especially in PPV cases (Figure 3). SF_6_ is 1 of 6 major greenhouse gases identified under the Kyoto Protocol and has a 100-year global warming potential 23 900 times greater than carbon dioxide.^18^ Its use accounted for 45% and 22% of total emissions in PPV and pneumatic retinopexy, respectively. In pneumatic retinopexy, a 1 cc syringe was flushed twice before the intravitreal injection of pure SF_6_. In contrast, a 50 cc syringe was flushed twice to ensure accurate dilution to 30% before injection during PPV. Although slight variation exists in the volume and number of flushes across different centers, 3 full flushes is standard practice. To mitigate the environmental impact of SF_6_ in PPV, some studies suggest the use of air tamponade as an alternative, providing evidence for efficacy while significantly reducing the carbon footprint of RRD repair.^19,20^

Alternatively, reevaluating intraoperative gas preparation processes and the choice of tamponading agent may warrant further consideration. Comparable fluorinated gases such as hexafluoromethane and octafluoropropane, which differ in expansile properties and duration of action, have a smaller carbon footprint with a 100-year global warming potential of 9200 and 7000, respectively.^13,20,21^ Although the selection of tamponade is influenced by various factors, a deliberate reduction of SF_6_ use in favor of air, hexafluoromethane, or octafluoropropane could substantially reduce carbon emissions.^21,22^

The greenhouse gas effects of modern anesthetic gases, such as sevoflurane, desflurane, isoflurane, and nitrous oxide, have been previously documented in the literature. Sevoflurane has the lowest 100-year global warming potential by a substantial margin and was exclusively used for maintenance of general anesthesia in scleral buckle cases.^14^ Adjusted sevoflurane use in cases involving general anesthesia accounted for 8% of the total emissions produced by scleral buckle; however, the use of other anesthetic agents could more than double the contribution of anesthetic gases. Several studies have recommended optimizing anesthetic protocols to use the minimum effective amount of gas required to maintain anesthesia, thereby reducing the associated economic cost and greenhouse gas emissions.^14,23^ Alternatively, when clinically appropriate, performing scleral buckle under sedation and local anesthesia can eliminate these emissions altogether.

Our study identified substantial differences in greenhouse gas emissions among the 3 procedures related to energy use. PPV and scleral buckle were performed in hospital-based operating rooms, while pneumatic retinopexy was conducted in an outpatient clinic. Operating rooms are a major source of greenhouse gas emissions in the healthcare system, and our study reiterates the carbon footprint of operating rooms.^10^

A limitation of this study is the exclusion of emissions related to patient transportation. Although our experience indicates that the number of follow-up visits is similar across PPV, pneumatic retinopexy, and scleral buckle procedures, follow-up protocols may vary at other centers. For example, the PIVOT study suggests that pneumatic retinopexy may require 1 additional follow-up visit compared to PPV over 12 months (10.8 vs 9.6).^6^

Based on comparable patient demographics and geographic factors between our study population and that of Power et al,^24^ who examined the travel-related carbon footprint of intravitreal injections, we estimated travel-related emissions to be 115.39 kg and 104.90 kg of carbon dioxide equivalents for pneumatic retinopexy and PPV, respectively. Notably, the PIVOT study reported differing primary anatomical success rates between pneumatic retinopexy and PPV (80.8% vs 93.2%) over 12 months, indicating that some patients required a secondary vitrectomy—a factor not accounted for in our study.^3,6,25^ Incorporating this would potentially increase the total greenhouse gas emission for pneumatic retinopexy to 7.34 kg carbon dioxide equivalents per case.

There is a substantial difference in greenhouse gas emissions among primary PPV, pneumatic retinopexy, and scleral buckle. This study quantifies and compares the environmental impact of each procedure and identifies the steps in the life cycle of each procedure that can be targeted to mitigate greenhouse gas emissions.

Practice patterns and surgical considerations for RRD repair are complex. Patient-centered factors and optimal patient outcomes should always be the primary goal. However, surgeon experience, preference, operating room availability, and economic and environmental considerations also influence decision-making. In cases of clinical equipoise, the broader environmental impact should be among surgical considerations, especially as we endeavor to reduce the overall carbon footprint of ophthalmic surgery.

## Supplemental Material

sj-docx-1-vrd-10.1177_24741264251367120 – Supplemental material for Carbon Footprint of Pars Plana Vitrectomy, Pneumatic Retinopexy, and Scleral Buckle Procedures for Rhegmatogenous Retinal Detachment Repair Supplemental material, sj-docx-1-vrd-10.1177_24741264251367120 for Carbon Footprint of Pars Plana Vitrectomy, Pneumatic Retinopexy, and Scleral Buckle Procedures for Rhegmatogenous Retinal Detachment Repair by Rahul Moorjani, Graeme Kenneth Loh, Matthew T.S. Tennant, Peter J. Kertes and Parampal S. Grewal in Journal of VitreoRetinal Diseases
