# Supplementary material for: Carbon Footprint of Pars Plana Vitrectomy, Pneumatic Retinopexy, and Scleral Buckle Procedures for Rhegmatogenous Retinal Detachment Repair
Source: J Vitreoretin Dis. 2025 Sep 3;9(6):836–41. doi: 10.1177/24741264251367120 (PMC12408532; doi:10.1177/24741264251367120)
Supplement: Supplementary file 1 [file sj-docx-1-vrd-10.1177_24741264251367120.docx]

| PPV | SB | PR |
| --- | --- | --- |
| Betadine (25 mL) | Betadine (25 mL) | Betadine (25 mL) |
| Sulfur Hexafluoride (150 mL) | Sulfur Hexafluoride (3 mL) | Sevoflurane gas (7.6 mL) |
| Applicators (1 pack) | Applicators (1 pack) | Applicators (1 pack) |
| IV tubing (1) | IV tubing (1) | Gloves (1 pair) |
| Oxygen tubing (1) | Oxygen tubing (1) | Drape (1) |
| Gloves (2 pairs) | Gloves (2 pairs) | Syringe (2) |
| Drapes (2) | Drapes (2) | Needles (2) |
| Sponges (6 pack) | Sponges (6 pack) | Sponges (6 pack) |
| Gown with disposable towel (2) | Gown with disposable towel (2) | Cannula (1) |
| Syringe (4) | Syringe (4) | Medication bottle (1) |
| Needles (7) | Needles (7) | Eye pad (2) |
| Cannula (1) | Cannula (1) | Balanced salt solution (15 mL) |
| Disposable tray (1) | Disposable tray (1) | Plastic garbage bag (1) |
| Medication bottles (2) | Medication bottles (6) |  |
| Eye pad (2) | Eye pad (2) |  |
| Eye shield | Eye shield |  |
| Balanced salt solution (500 mL) | Balanced salt solution (30 mL) |  |
| Table cover (1) | Table cover (1) |  |
| Disposable wraps (2) | Disposable wraps (2) |  |
| Plastic garbage bag (1) | Plastic garbage bag (1) |  |
| Infusion cannula (1) | Scleral buckle band and sleeve (1) |  |
| Vitrector handpiece (1) | Sutures (3) |  |
| Endoilluminator (1) |  |  |
| Trochars (3) |  |  |
| Venting cannula (1) |  |  |
| Vitrectomy cassette (1) |  |  |

**Supplementary table 1.** Comprehensive list of materials (with quantities) used in pars plana vitrectomy (PPV), scleral buckle (SB), and pneumatic retinopexy (PR).
